# Supplementary material for: Allele-specific endogenous tagging and quantitative analysis of β-catenin in colorectal cancer cells
Source: eLife. 2022 Jan 11;11:e64498. doi: 10.7554/eLife.64498 (PMC8752093; doi:10.7554/eLife.64498)
Supplement: Figure 2—source data 1. — (B) Cell lysates of indicated HCT116 cell lines analyzed by Western blotting with a β-catenin antibody; β-actin served as a loading control. (C) HCT116 β-cateninWTClover/∆45Cherry (clone #37) immunoprecipitation with GFP, Cherry and control beads, or with a β-catenin antibody followed by immunoblotting with indicated antibodies. Representative results from three independent experiments are shown. Figure 2—figure supplement 1: Validation of endogenously fluorescent-tagged β-catenin in HCT116 colon cancer cells. (A) HCT116 β-cateninWTClover/∆45Cherry (clone #37) cells express comparable amounts of β-catenin to the parental HCT116 WT cells. (B–D) Immunoprecipitation using HCT116 β-cateninWTClover/∆45 (clone #33 – left), β-cateninWT/∆45Cherry (clone #45 – middle), and β-cateninWTClover/∆45 (clone #24 – right) were performed with GFP/Cherry or control beads, followed by Western blotting with the indicated antibodies. Representative results from three independent experiments are shown. [file elife-64498-fig2-data1.zip › Figure 2Source Data 1.pdf]

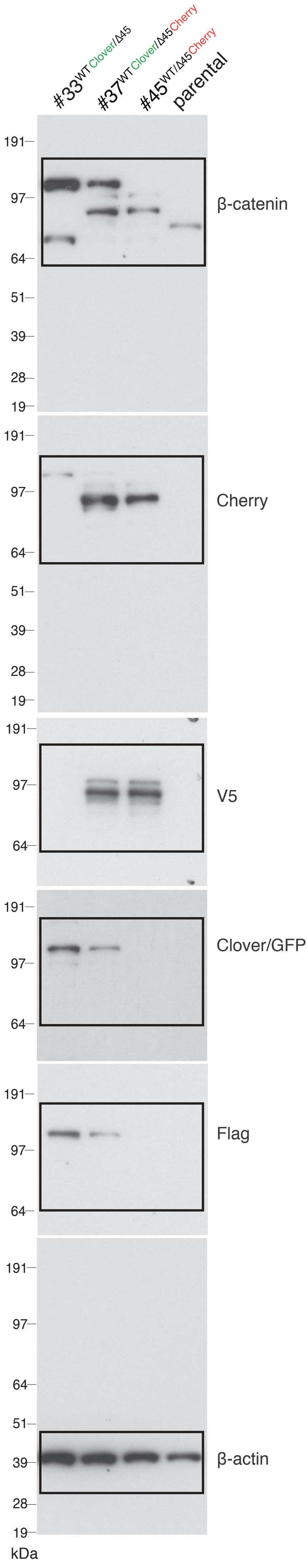

Figure 2: Identification and confirmation of tagged β-catenin alleles.
(B) Cell lysates of indicated HCT116 cell lines analysed by western blotting with a β-catenin antibody; β-actin served as a loading control. Representative results from three independent experiments are shown.

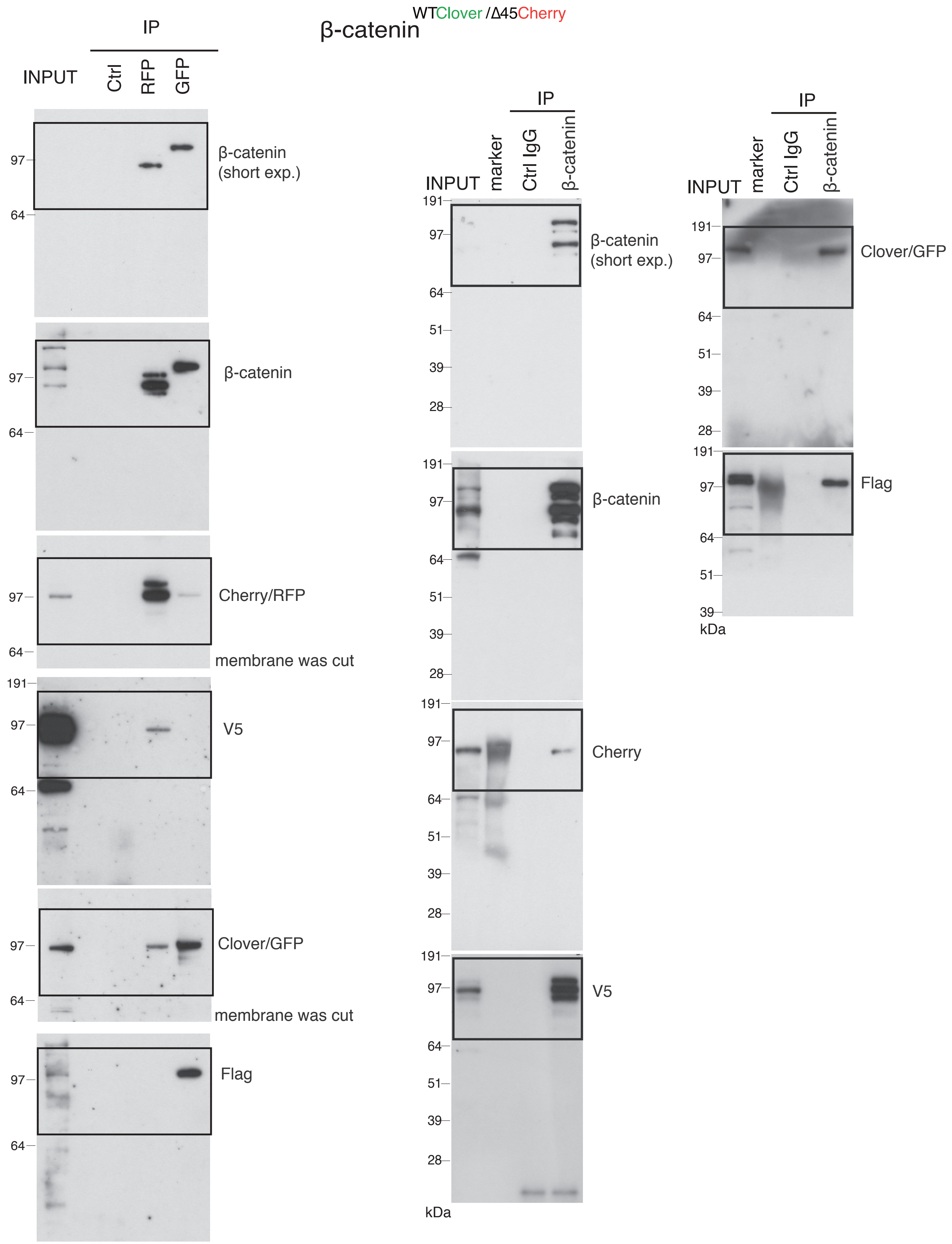

Figure 2: Identification and confirmation of tagged β-catenin alleles.

(C) HCT116 β-catenin$^{WTClover/\Delta45Cherry}$ (clone #37) immunoprecipitation with GFP/Clover, RFP/Cherry and control beads or with a β-catenin antibody followed by immunoblotting with indicated antibodies. Representative results from three independent experiments are shown.

The same samples were used for Figure 2C and S7B.

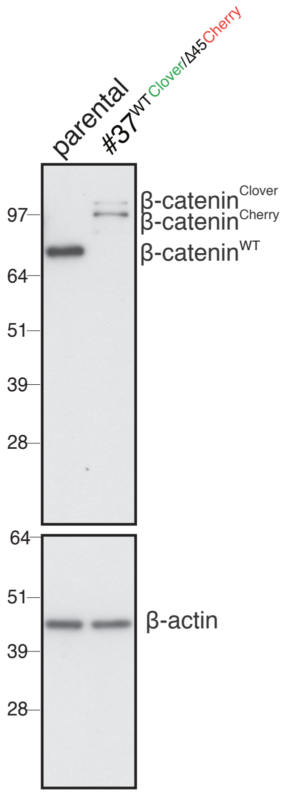

Figure 2-figure supplement 1: Validation of endogenously fluorescent-tagged β-catenin in HCT116 colon cancer cells.
(A) HCT116 β-catenin$^{WTClover/\Delta45Cherry}$ (clone #37) cells express comparable amounts of β-catenin to the parental HCT116 wild-type cells.

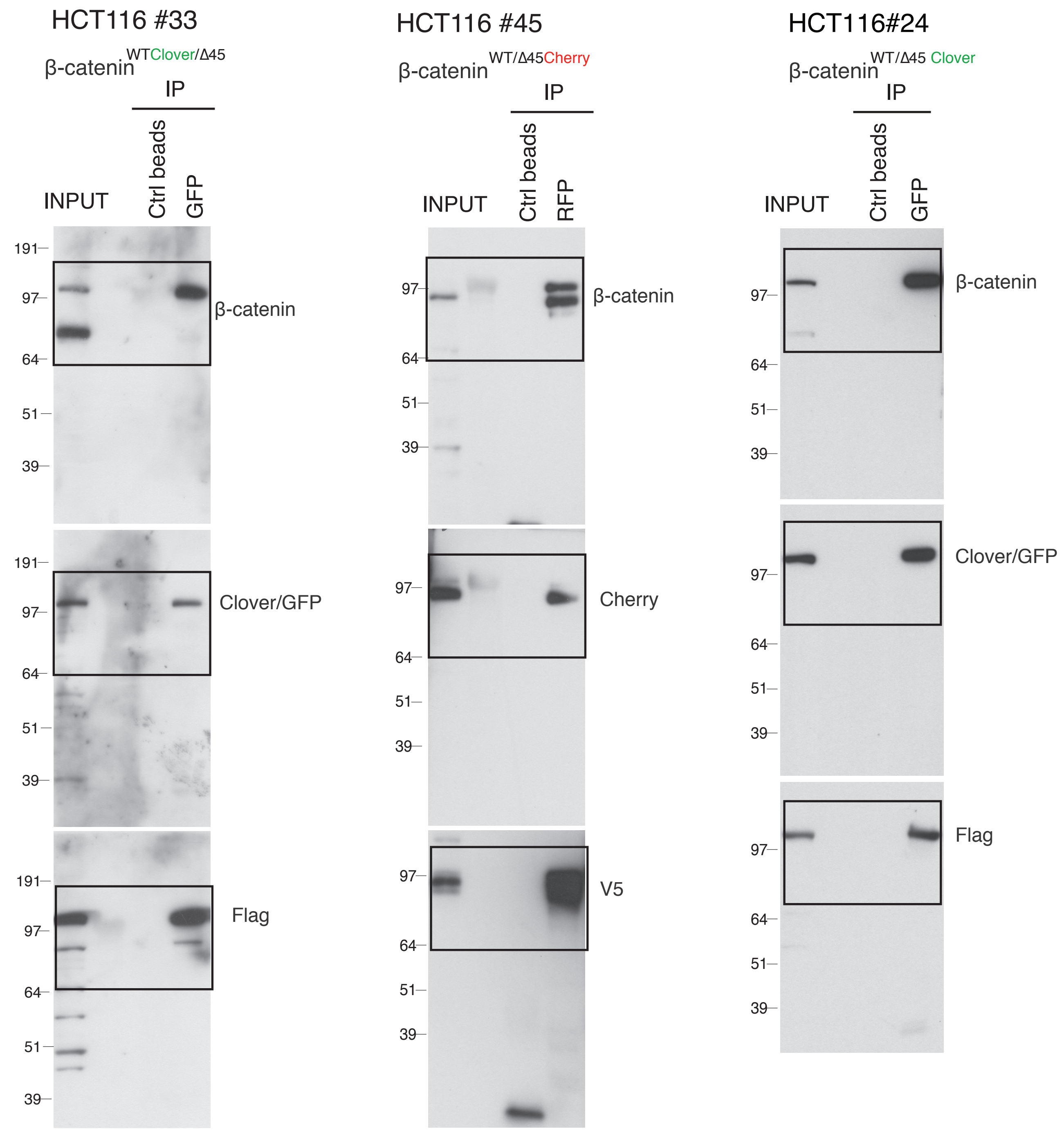

Figure 2-figure supplement 1: Validation of endogenously fluorescent-tagged β-catenin in HCT116 colon cancer cells.
(B,C,D) Immunoprecipitation using HCT116 β-catenin$^{WTClover/\Delta45}$ (clone #33 - B), β-catenin$^{WT/\Delta45Cherry}$ (clone #45 - C), β-catenin$^{WTClover/\Delta45}$ (clone #24 - D) were performed with control, GFP/Clover or RFP/Cherry beads, followed by western blotting with the indicated antibodies. Representative results from three independent experiments are shown.
