# Supplementary material for: Allele-specific endogenous tagging and quantitative analysis of β-catenin in colorectal cancer cells
Source: eLife. 2022 Jan 11;11:e64498. doi: 10.7554/eLife.64498 (PMC8752093; doi:10.7554/eLife.64498)
Supplement: Figure 3—source data 2. — (E) Immunoprecipitation of HCT116 clone β-cateninWTClover/∆45Cherry with E-cadherin confirms its interaction with β-catenin. Representative results from three independent experiments are shown. Figure 3—figure supplement 1: Validation of the physiological function of fluorescently tagged β-catenin. (C) Immunoprecipitation of HCT116 β-cateninWTClover/∆45 and β-cateninWT/∆45Cherry with E-cadherin validates its interaction with β-catenin. A representative immunoblot is displayed. [file elife-64498-fig3-data2.zip › Figure 3Source Data 2.pdf]

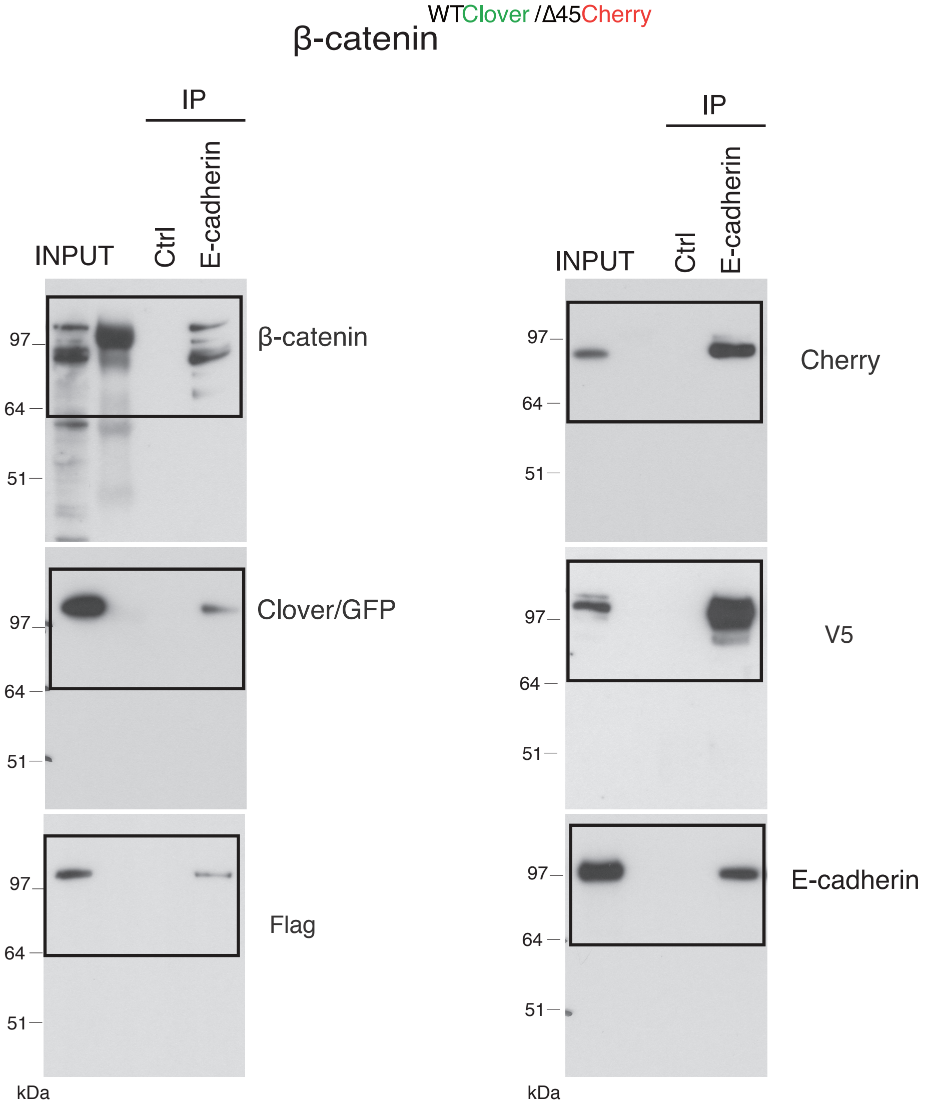

Figure 3. Fluorescently tagged β-catenin variants are functional and localize to adherens junctions. **(E)** Immunoprecipitation of HCT116 clone β-catenin$^{WTClover/\Delta45Cherry}$ with E-cadherin confirms its interaction with β-catenin. Representative results from three independent experiments are shown.

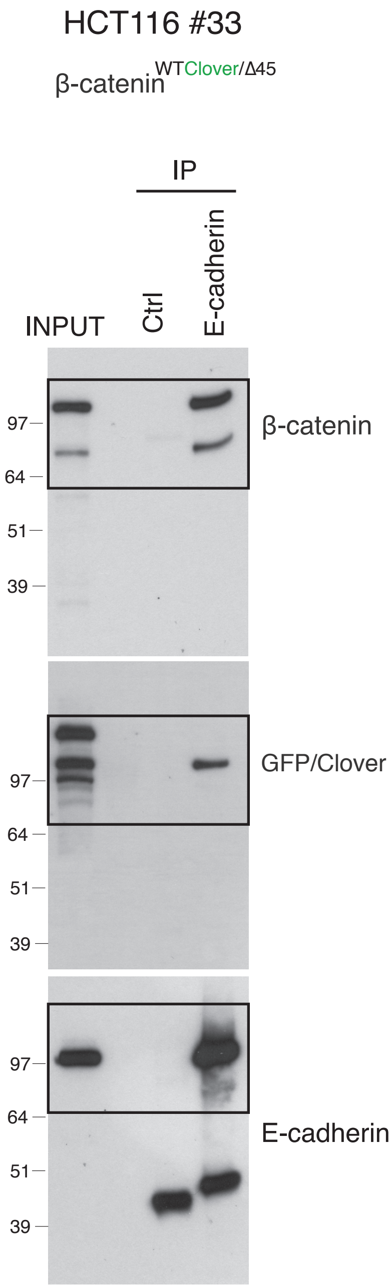

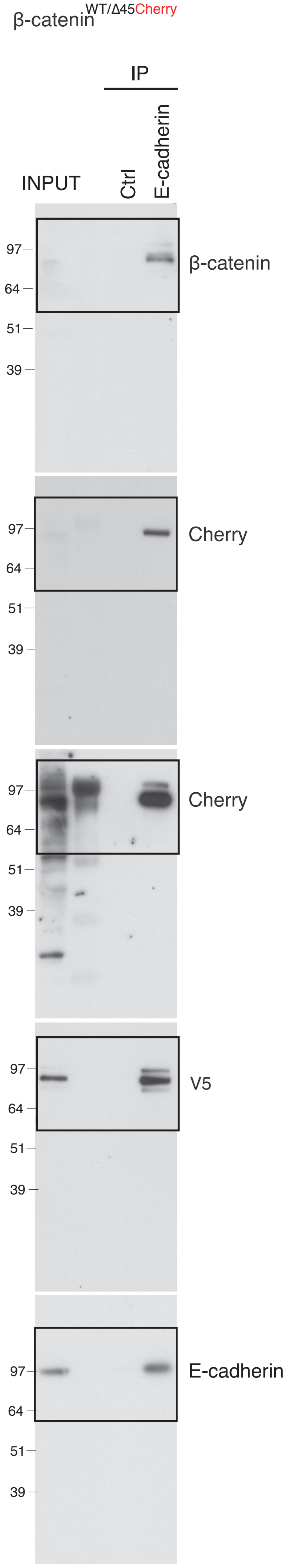

Figure 3-figure supplement 1: Validation of the physiological function of fluorescently tagged β-catenin.
(C) Immunoprecipitation of HCT116 β-catenin$^{WTClover/\Delta45}$ and β-catenin$^{WT/\Delta45Cherry}$ with E-cadherin validates its interaction with β-catenin. Representative immunoblots are displayed.
