# Supplementary material for: Allele-specific endogenous tagging and quantitative analysis of β-catenin in colorectal cancer cells
Source: eLife. 2022 Jan 11;11:e64498. doi: 10.7554/eLife.64498 (PMC8752093; doi:10.7554/eLife.64498)
Supplement: Figure 7—source data 2. — Figure 7—figure supplement 1: (B) Both β-catenin alleles of HCT116 cells bind to APC, GSK3β, and Axin1. IP with anti-APC antibody was performed GFP/Clover and Cherry were detected (left panel). IPs with RFP/Cherry and GFP/Clover beads were performed and β-catenin, Axin1, and GSK3β were detected (right panel). Representative experiments from three independent are shown. [file elife-64498-fig7-data2.zip › Figure 7XXXSource Data 2.pdf]

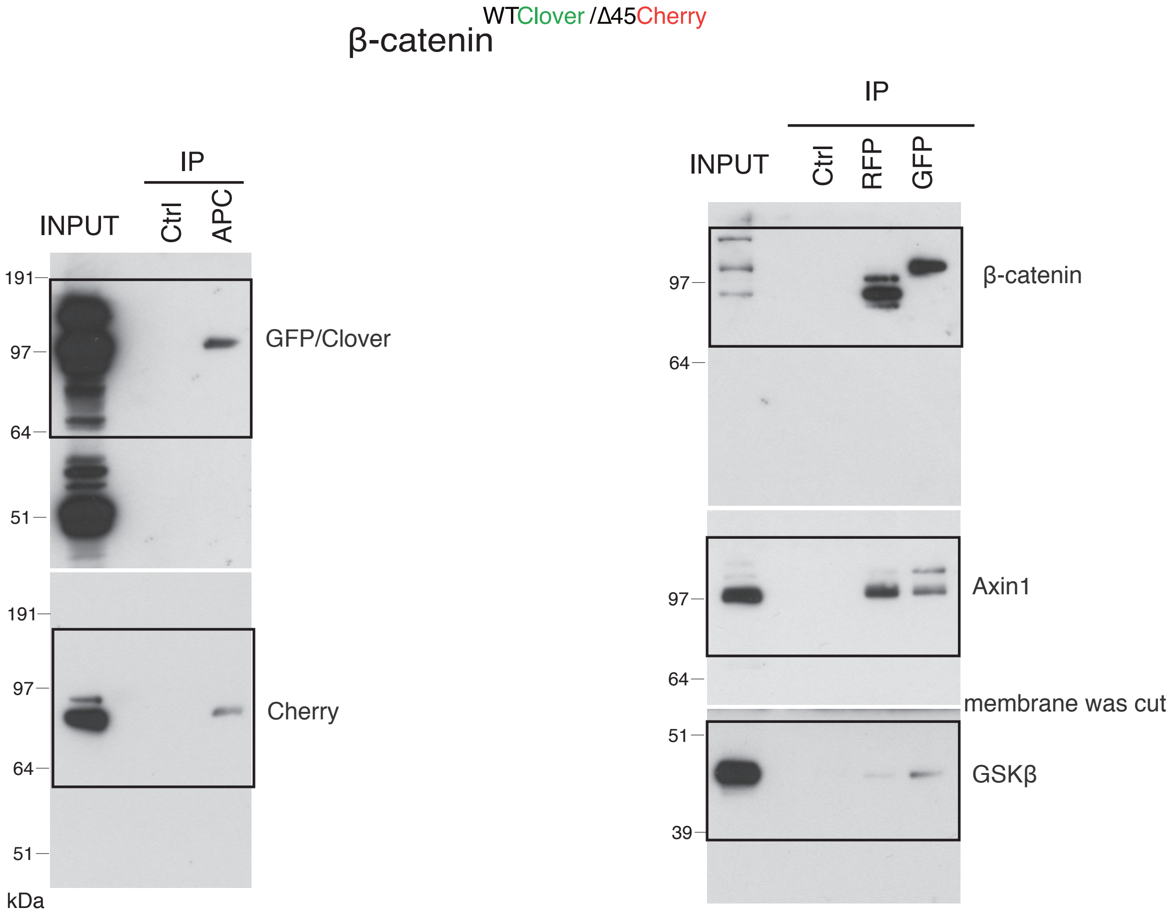


**Figure 7-figure supplement 1: Truncation of APC affects abundance and diffusion of wild-type but not mutant β-catenin in the nucleus.**

**(B)** Both β-catenin alleles of HCT116 cells bind to APC, GSK3β and Axin1. IP with anti-APC antibody was performed GFP/Clover and Cherry were detected (left panel). IPs with RFP/Cherry and GFP/Clover beads were performed and β-catenin, Axin1 and GSK3β were detected (right panel). Representative experiments from 3 independent are shown.
The same samples were used for Figure 2C.
