# Supplementary material for: Allele-specific endogenous tagging and quantitative analysis of β-catenin in colorectal cancer cells
Source: eLife. 2022 Jan 11;11:e64498. doi: 10.7554/eLife.64498 (PMC8752093; doi:10.7554/eLife.64498)
Supplement: Supplementary file 1. [file elife-64498-supp1.docx]

**Supplementary File 1**

| **Figure** | **Condition 1** | **Condition 2** | ***p*-value** | **star** |
| --- | --- | --- | --- | --- |
| 6A concentration | WT | Δ45 | 3.5E-13 | *** |
| 6A diffusion | WT | Δ45 | 9.1E-10 | *** |
| 6C concentration in cytoplasm | WT/DMSO | WT/CHIR99021 | 2.6E-19 | *** |
|  | Δ45/DMSO | Δ45/CHIR99021 | 0.0064 | ** |
| 6C diffusion in cytoplasm | WT/DMSO | WT/CHIR99021 | 3.1E-17 | *** |
|  | Δ45/DMSO | Δ45/CHIR99021 | 0.15 | NS |
| 6C concentration in nucleus | WT/DMSO | WT/CHIR99021 | 3.3E-22 | *** |
|  | Δ45/DMSO | Δ45/CHIR99021 | 5.0E-06 | *** |
| 6C diffusion in nucleus | WT/DMSO | WT/CHIR99021 | 2.7E-13 | *** |
|  | Δ45/DMSO | Δ45/CHIR99021 | 0.016 | * |
| 6D concentration in cytosol | WT/DMSO | WT/DMSO +Wnt3a | 0.026 | * |
|  |  | WT/LGK974 | 1.4E-17 | *** |
|  | Δ45/DMSO | Δ45/DMSO +Wnt3a | 0.34 | NS |
|  |  | Δ45/LGK974 Control | 4.4E-04 | *** |
|  | WT/LGK974 | WT/LGK974 +Wnt3a | 3.2E-13 | *** |
|  | Δ45/LGK974 | Δ45/LGK974 +Wnt3a | 0.031 | * |
| 6D diffusion in cytosol | WT/DMSO | WT/DMSO +Wnt3a | 8.5E-05 | *** |
|  |  | WT/LGK974 | 2.7E-11 | *** |
|  | Δ45/DMSO | Δ45/DMSO +Wnt3a | 0.801 | NS |
|  |  | Δ45/LGK974 Control | 6.0E-04 | *** |
|  | WT/LGK974 | WT/LGK974 +Wnt3a | 1.4E-11 | *** |
|  | Δ45/LGK974 | Δ45/LGK974 +Wnt3a | 0.030 | * |
| Figure 6-figure supplement 1B concentration in nucleus | WT/DMSO | WT/DMSO +Wnt3a | 0.0060 | ** |
|  |  | WT/LGK974 | 3.1E-18 | *** |
|  | Δ45/DMSO | Δ45/DMSO +Wnt3a | 0.17 | NS |
|  |  | Δ45/LGK974 Control | 9.2E-08 | *** |
|  | WT/LGK974 | WT/LGK974 +Wnt3a | 9.2E-13 | *** |
|  | Δ45/LGK974 | Δ45/LGK974 +Wnt3a | 0.0011 | ** |
| Figure 6-figure supplement 1B diffusion in nucleus | WT/DMSO | WT/DMSO +Wnt3a | 1.7E-04 | *** |
|  |  | WT/LGK974 | 1.2E-12 | *** |
|  | Δ45/DMSO | Δ45/DMSO +Wnt3a | 0.0018 | ** |
|  |  | Δ45/LGK974 Control | 0.13 | NS |
|  | WT/LGK974 | WT/LGK974 +Wnt3a | 4.1E-13 | *** |
|  | Δ45/LGK974 | Δ45/LGK974 +Wnt3a | 5.0E-06 | *** |
| 7B concentration in cytoplasm | WT/DMSO | WT APC^LOF^ /DMSO | 3.6E-18 | *** |
|  | Δ45/DMSO | Δ45 APC^LOF^/DMSO | 0.048 | * |
|  | WT APC^LOF^ /DMSO | WT APC^LOF^ /CHIR99021 | 4.5E-04 | *** |
| 7B diffusion in cytoplasm | WT/DMSO | WT APC^LOF^ /DMSO | 2.5E-16 | *** |
|  | WT/DMSO | WT APC^LOF^/DMSO | 0.0016 | ** |
|  | WT APC^LOF^ /DMSO | WT APC^LOF^ /CHIR99021 | 0.0047 | ** |
| Figure 7-figure supplement 1A concentration in nucleus | WT/DMSO | WT APC^LOF^ /DMSO | 1.9E-23 | *** |
|  | Δ45/DMSO | Δ45 APC^LOF^/DMSO | 0.99 | NS |
|  | WT APC^LOF^ /DMSO | WT APC^LOF^ /CHIR99021 | 0.0019 | ** |
| Figure 7-figure supplement 1A diffusion in nucleus | WT/DMSO | WT APC^LOF^ /DMSO | 1.8E-15 | *** |
|  | Δ45/DMSO | Δ45 APC^LOF^/DMSO | 0.38 | NS |
|  | WT APC^LOF^ /DMSO | WT APC^LOF^ /CHIR99021 | 0.081 | NS |
