# Supplementary material for: Allele-specific endogenous tagging and quantitative analysis of β-catenin in colorectal cancer cells
Source: eLife. 2022 Jan 11;11:e64498. doi: 10.7554/eLife.64498 (PMC8752093; doi:10.7554/eLife.64498)
Supplement: Supplementary file 2. [file elife-64498-supp2.docx]

**Supplementary File 2**

**siRNAs**

| Target  gene | Supplier | Catalogue # | Sequence |
| --- | --- | --- | --- |
| UBC | GE Healthcare  Dharmacon/Horizon | MU-019408-01-0002 | #1 GTGAAGACCCTGACTGGTA  #2 AAGCAAAGATCCAGGACAA  #3 GAAGATGGACGCACCCTGT  #4 GTAAGACCATCACTCTCGA |
| Non targeting | GE Healthcare  Dharmacon/Horizon | D‐001810‐02 | UGGUUUACAUGUUGUGUGA |
| Control | Ambion | S29712 |  |
| GFP | GE Healthcare  Dharmacon//Horizon | D-001300-01-05 | GCAAGCTGACCCTGAAGTTC |
| CTNNB1 | Ambion | S438 | CUGUUGGAUUGAUUCGAAAtt |
| Cherry | IDT | Custom design | #3 rCrArU rGrGrC rCrArU rCrArU  rCrArA rGrGrA rGrUrU rCrArU rG |
