# Supplementary material for: Allele-specific endogenous tagging and quantitative analysis of β-catenin in colorectal cancer cells
Source: eLife. 2022 Jan 11;11:e64498. doi: 10.7554/eLife.64498 (PMC8752093; doi:10.7554/eLife.64498)
Supplement: Supplementary file 3. [file elife-64498-supp3.docx]

**Supplementary File 3**

**Antibodies**

| Antibody | Company | Catalogue # | Species | Dilution |
| --- | --- | --- | --- | --- |
| β-actin HRP | Santa Cruz Biotechnology | 47778 | rabbit | 1:20000 |
| β-actin | Santa Cruz Biotechnology | 47778 | rabbit | 1:40000 |
| β-catenin | Dianova/Affinity BioReagent | MA1-2001 | mouse | 1:3000 |
| Cherry | Clontech | 632543 | mouse | 1:1000 |
| GFP | Invitrogen | 332600 | mouse | 1:1000 |
| GFP | Invitrogen | A6455 | rabbit | 1:1000 |
| E-Cadherin | BD Biosciences | 610182 | mouse | 1:1000 |
| V5 | Rockland | 600-401-378 | rabbit | 1:2000 |
| V5 | Thermo Scientific | 15253 | mouse | 1:1000 |
| Flag | Sigma | F7425 | rabbit | 1:1000 |
| Flag | Sigma | F3165 | mouse | 1:1000 |
| APC ALI 12-28 | Santa Cruz Biotechnology | sc-53165 | mouse | IP |
| Axin1 C76H11 | Cell Signaling Technology | 2087 | rabbit | 1:1000 |
| GSK3β D5C5Z | Cell Signaling Technology | 12456 | rabbit | 1:1000 |
| Normal IgG | Cell Signaling Technology | 2729 | rabbit |  |
| IgG1 K isotype control | eBioscience | 16-4714-81 | mouse |  |
| Anti-mouse IgG-HRP | Jackson ImmunoResearch | 115-035-003 | Goat | 1:10000 |
| Anti-rabbit IgG-HRP | Jackson ImmunoResearch | 111-035-003 | Goat | 1:10000 |
| True Blot ULTRA Anti-mouse IgG-HRP | [eBioscience](https://b110-sv4.inet.dkfz-heidelberg.de/Labcollector/Heidelberg/admin.php?action=sellers) | 18-8817-33 |  | 1:2500-1:5000 |
