# Supplementary material for: Allele-specific endogenous tagging and quantitative analysis of β-catenin in colorectal cancer cells
Source: eLife. 2022 Jan 11;11:e64498. doi: 10.7554/eLife.64498 (PMC8752093; doi:10.7554/eLife.64498)
Supplement: Supplementary file 4. [file elife-64498-supp4.docx]

**Supplementary File 4**

**Plasmids**

| Plasmid | Reference | Comments |
| --- | --- | --- |
| Actin-Renilla luciferase | [(Nickles et al., 2012)](https://paperpile.com/c/mjtR2X/yDOi) | TCF4/Wnt reporter assay |
| TCF4/Wnt-reporter | [(Demir et al., 2013)](https://paperpile.com/c/mjtR2X/y9Pu) | TCF4/Wnt reporter assay |
| px459sgCTNNB1 | This study | sgRNA:  TGACCTGTAAATCATCCTTT |
| KOZAK_FLAG_mClover3 | This study | Overexpression plasmid |
| KOZAK_V5_mCherry | This study | Overexpression plasmid |
| px459sgAPC#b | This study | sgRNA: TAGAACCAAATCCAGCAGA |
| pSpCas9(BB)-2A-GFP (PX458) | Addgene #48138 | Control vector |
| pMK-RQ HA-FLAG-mClover-PGK-HygRHA | This study | Donor template |
| pMK-RQ HA-V5-mCherry-PGK-BRS-HA | This study | Donor template |
