# Supplementary material for: Allele-specific endogenous tagging and quantitative analysis of β-catenin in colorectal cancer cells
Source: eLife. 2022 Jan 11;11:e64498. doi: 10.7554/eLife.64498 (PMC8752093; doi:10.7554/eLife.64498)
Supplement: Supplementary file 5. [file elife-64498-supp5.docx]

**Supplementary File 5**

**Primers and Roche UPL index used in qPCR experiments**

| Gene | UPL Probe # | Forward (5’-3’) | Reverse (5’-3’) |
| --- | --- | --- | --- |
| Clover | 148 | TATATCACGGCCGACAAGC | GTTGTGGCGGATCTTGAAGT |
| Cherry | 152 | GTGACCGTGACCCAGGAC | GTGACCGTGACCCAGGAC |
| AXIN2 | 88 | AGAGCAGCTCAGCAAAAAGG | CCTTCATACATCGGGAGCAC |
| GAPDH | 60 | AGCCACATCGCTCAGACAC | GCCCAATACGACCAAATCC |
| UBC | 11 | ACCAGCAGAGGCTGATCTTT | TCTGGATGTTGTAGTCTGACAGG |
| CTNNB1 | 21 | AGCTGACCAGCTCTCTCTTCA | CCAATATCAAGTCCAAGATCAGC |
