# Supplementary material for: Allele-specific endogenous tagging and quantitative analysis of β-catenin in colorectal cancer cells
Source: eLife. 2022 Jan 11;11:e64498. doi: 10.7554/eLife.64498 (PMC8752093; doi:10.7554/eLife.64498)
Supplement: Supplementary file 6. [file elife-64498-supp6.docx]

**Supplementary File 6**

**Oligonucleotides**

| Name | Primer Sequence (5’-3’) |
| --- | --- |
| 3XFLAG_F | AGCTGGCCTGGTTTGATACTGACCTGGACTATAAGGACCACGACGGAGACTA |
| 3XFLAG_R | CGATCCGCCACCGCCAGAGCCACCTCCGCCTGAACCGCCACCACCTGACTTATCGTCATCGTCTTTGTAATCAATATC |
| GFP_R | CCCAAACTGGCTTTTTAAAACTTCTTTACTTGTACAGCTCGTCCATGCCGAGAGTGA |
| GFP_F | TCAGGTGGTGGCGGTTCAGGCGGAGGTGGCTCTGGCGGTGGCGGATCGCCGGTCGCCACCATGGTGAGCAAGG |
| RHA2_F | CGGCGGCATGGACGAGCTGTACAAGAGAAGTTTTAAAAAGCCAGTTTGGG |
| RHA2_R | CTATGACCATGATTACGCCAAGCTTTTCTTGAGTCACTCCCAAAATCCA |
| CHERRY 2_R | CCCAAACTGGCTTTTTAAAACTTCTCTTGTACAGCTCGTCCATGCCG |
| CHERRY2_F | TCAGGTGGTGGCGGTTCAGGCGGAGGTGGCTCTGGCGGTGGCGGATCGATGGTGAGCAAGGGCGAGGA |
| V5_2_R | CGATCCGCCACCGCCAGAGCCACCTCCGCCTGAACCGCCACCACCTGACGTAGAATCGAGACCGA |
| V5_2_F | GCTGGCCTGGTTTGATACTGACCTGGGTAAGCCTATCCCTAACC |
| LHA2_R | GGGATAGGCTTACCCAGGTCAGTATCAAACCAGGCCAG |
| LHA2_F | GTTGTAAAACGACGGCCAGTGAATTAAGCTTGACAGGGGTAAATGCAGAGTGTTCAG |
| PGK_REV | CCTCCCCTACCCGGTAGAATTT |
| BCAT_GDNA_R | AACAAGCAAGGCTAGGGTTT |
| BCAT_CHERRY_R | AGCCGTACATGAACTGAGG |
| BCAT_CHERRY_F | AGCCGTACATGAACTGAGG |
| BCAT_GDNAF | TCACTGCCTAGGTGGTCACA |
| BCAT_GFPF | ACCACTACCAGCAGAACAC |
| BCAT_GFPR | TCGTGCTGCTTCATGTGGT |
| SGBCAT_FSEQ | TGGCACCACTTCCCGC |
| SGBCAT_RSEQ | CTGTGGGCGAGGCCAT |
| BCAT_TAG2_FW | AAATAGTGCCTTTCCTTAGTAC |
| BCAT_TAG2_R | AATTAGTGTTCTACACCATTAC |
| ATG_BCAT1F | AGAAAAGCGGCTGTTAGTCACTG |
| ATG_BCAT2_F | TGTTAGTCACTGGCAGCAACAGTC |
| BCAT_TAG3_F | AGCAACAGTCTTACCTGGACTCTG |
| BCAT_ATG3_R | GCATCTGTGATGGTTCAGCCAAA |
| BCAT-TAG3_F | ATGAAACCACTAAAGCGCAGATTC |
| BCAT_TAG3_R | TCTTGAAGCATCGTATCACAGCAG |
| CLOVER_R | CGTTGTGGCGGATCTTGAAGTT |
| CLOVER_F | AACTTCAAGATCCGCCACAACG |
| CHERRY_MID_R | CCATGGTACGACACTGCATT |
| CHERRY_MID_F | AATGCAGTGTCGTACCATGG |
| CTNNB_afterHA_r | AGGCCAATCACAATGCAAGT |
| insert_rev | GGGGGAACTTCCTGACTAGG |
| BCATC-term_f_Seq | TATGGACCCCATGATGGAAC |
| BRS_f | TGCAGTTTCGAATGGACAAA |
| CTNNB1_afterHA_3rev | CTCGACCAAAAAGGACCAGA |
| HGR_3_f | ATTTCGGCTCCAACAATGTC |
| CHERRY_3_F | AAGCAGAGGCTGAAGCTGAA |
| CTNNB1_Cterm_f | TTTGCCTTCCTTCTTGCCTA |
| Clover_end_for | GTCCAAGCTGAGCAAAGACC |
| Clover_mid_f | ACTTCAAGATCCGCCACAAC |
| Cherry_end_f | CACCTACAAGGCCAAGAAGC |
| BRS_rev | TACCAATCGCAATGGCTTCT |
| HGR_rev | GATGTTGGCGACCTCGTATT |
| HGR_2_rev | ACATTGTTGGAGCCGAAATC |
| BRS_2_f | TGTTTGTGCAGAAGCCATTG |
| HGR_2_f | TCAGCGAGAGCCTGACCTAT |
| HGR_f | GAATTCAGCGAGAGCCTGAC |
| Clover_end_r | GGTCTTTGCTCAGCTTGGAC |
| Cherry_end_r | GCTTCTTGGCCTTGTAGGTG |
| Cherry_mid2_for | GACATCCTGTCCCCTCAGTT |
| BRS_3_f | GCGACAGAGAAGATTACAATGC |
| BRS_3_rev | TTCGGGTATATTTGAGTGGAATG |
| BRS_4_f_seq | TTGATTCCCACTTTGTGGTTC |
| BRS_5_f_seq | TCTCTCGTGGGATCATTGTTT |
